# The effects of tourniquet use on blood loss in primary total knee arthroplasty for patients with osteoarthritis: a meta-analysis

**DOI:** 10.1186/s13018-019-1422-4

**Published:** 2019-11-08

**Authors:** D. F. Cai, Q. H. Fan, H. H. Zhong, S. Peng, H. Song

**Affiliations:** Department of Orthopaedic Surgery, Affiliated Hospital of Zunyi Medical College, 149 Dalian Road, Huichuan District, Zunyi City, Gui Zhou Province China

**Keywords:** Total knee arthroplasty, Tourniquet, Blood loss, Complications

## Abstract

**Background:**

The tourniquet is a common medical instrument used in total knee arthroplasty (TKA). However, there has always been a debate about the use of a tourniquet and there is no published meta-analysis to study the effects of a tourniquet on blood loss in primary TKA for patients with osteoarthritis.

**Methods:**

We performed a literature review on high-quality clinical studies to determine the effects of using a tourniquet or not on blood loss in cemented TKA. PubMed, Web of Science, MEDLINE, Embase, and the Cochrane Library were searched up to November 2018 for relevant randomized controlled trials (RCTs). We conducted a meta-analysis following the guidelines of the Cochrane Reviewer’s Handbook. We used the Cochrane Collaboration’s tool for assessing the risk of bias of each trial. The statistical analysis was performed with Review Manager statistical software (version 5.3).

**Results:**

Eleven RCTs involving 541 patients (541 knees) were included in this meta-analysis. There were 271 patients (271 knees) in the tourniquet group and 270 patients (270 knees) in the no tourniquet group. The results showed that using a tourniquet significantly decreased intraoperative blood loss (*P* < 0.002), calculated blood loss (*P* < 0.002), and the time of operation (*P* < 0.002), but tourniquet use did not significantly decrease postoperative blood loss (*P* > 0.05), total blood loss (*P* > 0.05), the rate of transfusion (*P* > 0.05), and of deep vein thrombosis (DVT) (*P* > 0.05) in TKA.

**Conclusions:**

Using a tourniquet can significantly decrease intraoperative blood loss, calculated blood loss, and operation time but does not significantly decrease the rate of transfusion or the rate of DVT in TKA. More research is needed to determine if there are fewer complications in TKA without the use of tourniquets.

## Introduction

The tourniquet is a common medical instrument used in total knee arthroplasty (TKA). A recent study of the American Association of Hip and Knee Surgeons found that approximately 95% of surgeons used tourniquets during TKA [1]. However, there has always been a debate about the pros and cons of tourniquet use [2]. For supporters, the tourniquet has several advantages in TKA: (1) a tourniquet can provide a bloodless field of view for surgery, (2) a tourniquet may help reduce intraoperative blood loss and improve cement penetration, and (3) a tourniquet could also shorten the operation time [3]. The disadvantages of tourniquet use mainly include damaging blood vessels and local soft tissue and increasing fibrinolytic activity [4]. Although tranexamic acid can decrease fibrinolytic activity, when combined with the use of a tourniquet, fibrinolytic activity will increase [5]. A tourniquet can also lead to local tissue swelling or hypoxia, which then affects wound healing [6–8] and produces more pain in the immediate post-operative surgery [3, 9]. Applying a tourniquet to the quadriceps femoris can affect the intraoperative patellar tracking and disturb the surgeon’s judgment of this movement. Tourniquets are also thought to be associated with an increased risk of deep vein thrombosis (DVT), wound infection or poor healing, postoperative dysfunction, and increased blood loss [7]. Moreover, some studies found that the use of a tourniquet may increase postoperative hidden blood loss [10].

There is no agreement on the pros and cons of tourniquet use in TKA. Some researchers wanted to gather reliable evidence by integrating high-quality clinical trial data. However, their results were not convincing, because the included patients had different primary diseases, such as rheumatoid arthritis, osteoarthritis, and even patients with revision, and different primary diseases may contribute to the incidence of complications and produce bias [11].

For these reasons, the conclusions of these meta-analyses need further support. Additionally, there is no published meta-analysis to study the effects of a tourniquet on blood loss in primary TKA for patients with osteoarthritis. Therefore, we aimed to assess the effect of tourniquet use on reducing blood loss and to determine the possible risks of using a tourniquet in primary TKA for patients with osteoarthritis.

## Materials and methods

### Search strategy

Our research began on September 1, 2018. The electronic databases of PubMed, Web of Science, MEDLINE, Embase, and the Cochrane Library were screened up to November 2018. The following keywords were used in the search: “total knee arthroplasty,” “total knee replacement,” “TKA,” “TKR,” “tourniquet,” and “randomized controlled trial.” Boolean operators were used to combine the terms. Reference lists of the relevant papers, especially papers included in the published meta-analyses, were screened. No restriction was made for publication status or language.

### Study inclusion and exclusion criteria

This meta-analysis was based on the guidelines described in the Cochrane Handbook for Systematic Reviews of Interventions [12]. We included literature according to the following criteria: (1) randomized controlled trials (RCTs) that compared patients undergoing primary TKA with or without the use of a tourniquet and (2) trials in which the patients received TKA for osteoarthritis. The exclusion criteria were as follows: studies that included a different tourniquet application strategy or revision TKA, studies in which patients received TKA for rheumatoid arthritis, animal studies, and duplicate studies or data. Two of the authors (DF Cai and QH Fan) independently scanned the titles and abstracts of all relevant studies and selected the RCTs according to the inclusion and exclusion criteria. If there were discrepancies regarding study inclusion, a third reviewer (Song H) was consulted, and the disagreement was resolved through discussion.

### Data extraction

Two of the authors (DF Cai and QH Fan) independently extracted relevant data based on a well-designed data extraction format that contains manuscript information, participant demographics, clinical outcomes, and any recorded complications. The clinical outcomes included total blood loss; calculated blood loss, which was calculated by the methods described by Gross [13] and thought to represent the true loss of blood; postoperative blood loss; intraoperative blood loss; blood transfusion volume; and blood transfusion rate. Recorded complications included any kind of wound complications, muscle or nerve injury, DVT, and pulmonary embolism (PE).

Discrepancies on data extraction were resolved by consulting another investigator (Song H); if the authors failed to reach a consensus, discrepancies were resolved by group discussion.

### Risk of bias

Two independent researchers (DF Cai and QH Fan) assessed the risk of bias of each included RCT by using the risk assessment tool recommended by the Cochrane Reviewer’s Handbook 5.1.0 [14]. The assessment scale includes six domains: sequence generation, allocation concealment, blinding, incomplete outcome data, selective outcome reporting, and other potential threats to validity.

Any discrepancies in the risk assessment were resolved by consulting another investigator (Song H); if failing to reach a consensus, discrepancies were resolved by group discussion.

### Statistical analysis

The analysis was conducted using Review Manager statistical software (version 5.3, Copenhagen: The Nordic Cochrane Centre, The Cochrane Collaboration, 2014). For continuous outcome data, a weighted mean difference (WMD) and 95% confidence interval (CI) were calculated by means and standard deviations (SDs). For dichotomous outcomes, the risk ratio or relative risk (RR) and 95% CI were calculated as the summary statistics. Statistical heterogeneity was determined by the chi-square test and *I*^2^. A value of *I*^2^ < 25% indicated low statistical heterogeneity; 25% ≤ *I*^2^ < 50% indicated moderate statistical heterogeneity; and 50% ≤ *I*^2^ < 75% indicated high statistical heterogeneity [15]. *P* < 0.05 was considered to be statistically significant. A random-effects analysis was used to synthesize heterogeneous data, and a fixed-effects analysis was used to synthesize data when the data were not heterogeneous [16].

## Result

### Selection process of the included studies

**Fig. 1:**
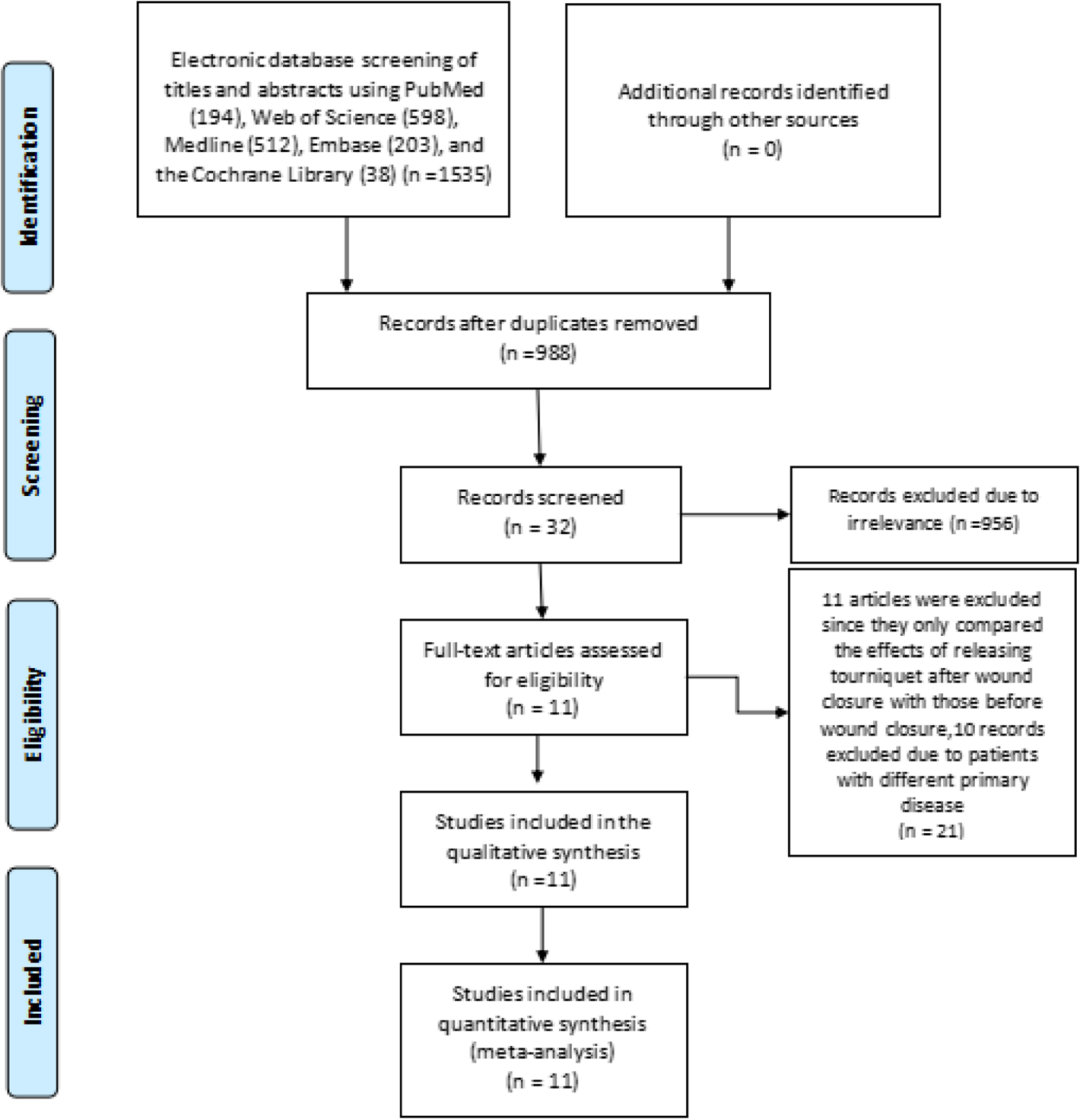
Flowchart showing the selection process of randomized controlled trials

By comprehensive review of the electronic databases, a total of 1535 articles were initially identified. Of these, 194 articles were from PubMed, 598 articles were from Web of Science, 512 articles were from MEDLINE, 203 articles were from Embase, and 28 articles were from the Cochrane Library. A total of 547 articles were excluded as duplicates, and 956 articles were excluded by scanning the titles and the abstracts. After reading the full texts, 11 articles were excluded since they only compared the effects of releasing tourniquet after wound closure with those before wound closure, and 10 articles were excluded because patients with different primary diseases were included. Finally, a total of 11 articles including 541 patients (541 knees) were included in this meta-analysis [17–27]. There were 271 patients (271 knees) in the tourniquet group and 270 patients (270 knees) in the no tourniquet group. A flowchart of RCT selection is shown in Fig. 1, and the characteristics of the included articles are shown in Table 1.

**Table 1:** Characteristics of the included articles

Study	Year	Sample size		Sex (M/F)		Mean age (years)		BMI (kg/m^2^)		Cemented	Prosthesis type	Patellar resurfacing	Thromboprophylaxis	Hemostasis
		T	NT	T	NT	T	NT	T	NT					
P. Aglietti	1999	10	10	3/7	4/6	70.0	68.0	27.9	27.3	Y	M.B.K, Zimmer	NS	NS	NS
H.M. Wakankar	1999	37	40	11/26	14/26	72.5	71.8	NS	NS	Y	Insall-Burstein	Y	Warfarin	NS
Eric Vanden	2002	40	40	9/31	16/24	72.5	68.5	NS	NS	Y	Wallaby I, Protek	NS	NS	NS
G. Matziolis	2005	10	10	2/8	3/7	72.4	76.6	28.3	29.5	Y	PFC Sigma, Depuy	N	NS	NS
K. Kageyama	2007	11	11	2/9	2/9	73.0	76.0	26.6	24.7	NS	NS	NS	NS	NS
Ta-Wei Tai	2012	36	36	9/27	8/28	72.1	71.5	28.6	27.9	Y	Genesis II or U2	NS	NS	NS
Hakan Ledin	2012	25	23	10/15	9/14	70.0	71.0	29.0	28.0	Y	NexgenCR, Zimmer	NS	Low-molecular-weight	NS
David Liu	2014	10	10	7/3	9/1	67.0	70.0	25.6	27.1	Y	NS	Y	NS	NS
Ashir Ejaz	2014	33	31	18/15	17/14	68.0	68.0	25.0	25.0	Y	NexgenCR, Zimmer	Y	Rivaroxaban	Tranexamic
Ashir Ejaz	2015	31	31	16/15	17/14	68.3	68.2	25.1	25.2	Y	NS	Y	NS	NS
A. Douglas	2016	28	28	16/12	16/12	62.0	62.0	29.0	29.0	Y	NS	N	Y	NS

*T* tourniquet, *NT* no tourniquet

### Risk of bias assessment

**Fig. 2:**
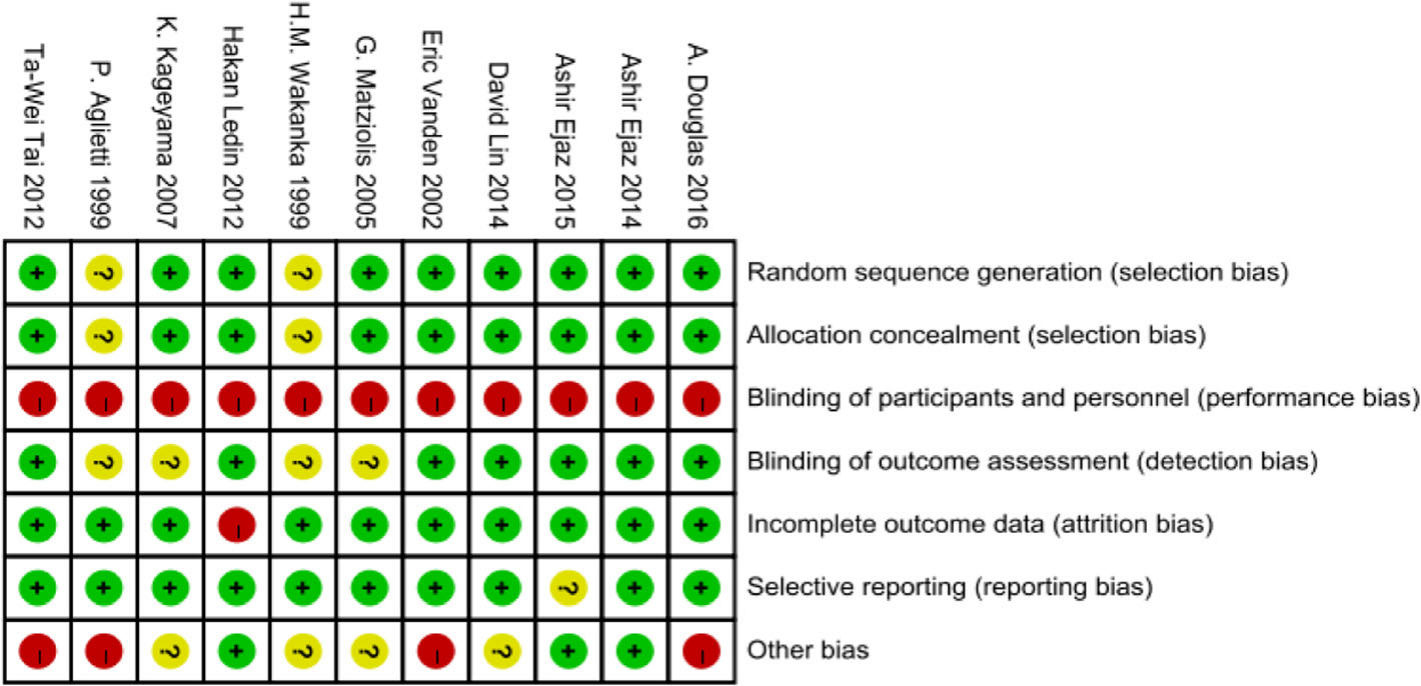
Risk of bias summary. Green indicates that the criterion is satisfied. Yellow indicates that it is unclear whether the criterion is satisfied or not. Red indicates that the study did not meet the criterion

In eight RCTs [17, 18, 20–22, 24–27] of those included, the methods of randomization were described, and the allocation of patients was concealed by using sealed envelopes. None of the studies blinded the surgeons. Seven RCTs [20–22, 24–27] involved blinding of the outcome assessments. All but one RCT [25] afforded complete outcome data, and none of the included RCTs exhibited selective outcome reporting. The results are summarized in Fig. 2.

### Clinical outcome measures

**Fig. 3:**
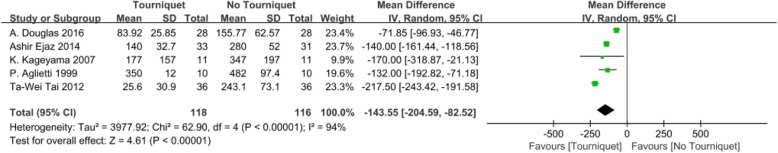
Forest plot for intraoperative blood loss

Among the included studies, five [17, 19, 21, 22, 27] provided data on intraoperative blood loss. There was a significant difference between groups in terms of intraoperative blood loss, and the pooled data showed that application of a tourniquet decreased intraoperative blood loss by 143.55 mL (WMD = − 143.55 mL; 95% CI, 204.59 to − 82.52; *n* = 234; *P* < 0.00001; *I*^2^ = 94%), as shown in the forest plot (Fig. 3).

**Fig. 4:**
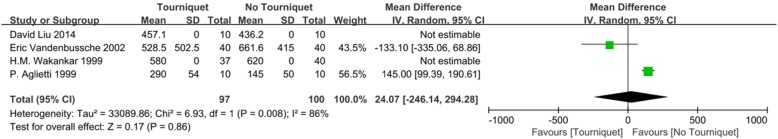
Forest plot for postoperative blood loss

Four of the included studies [19, 24–26] reported the volume of postoperative blood loss. Postoperative blood loss was measured by calculating the postoperative blood drainage volumes. Pooled data showed no significant difference between the groups in terms of postoperative blood loss (WMD = 24.07 mL; 95% CI, − 246.14 to 294.28; *n* = 197; *P* = 0.86; *I*^2^ = 86%), as shown in Fig. 4.

**Fig. 5:**
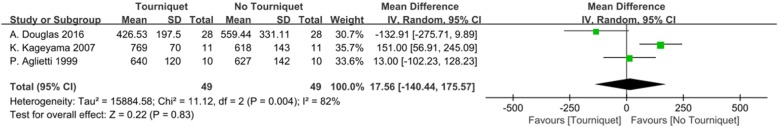
Forest plot for total blood loss

Three studies [17, 19, 21] reported data on total blood loss. Total blood loss means the sum of intraoperative and postoperative blood loss. No significant difference was observed between the tourniquet and no tourniquet groups in terms of postoperative blood loss (WMD = 17.56 mL; 95% CI, − 140.44 to 175.57; *n* = 98; *P* = 0.83; *I*^2^ = 82%), as shown in Fig. 5.

**Fig. 6:**
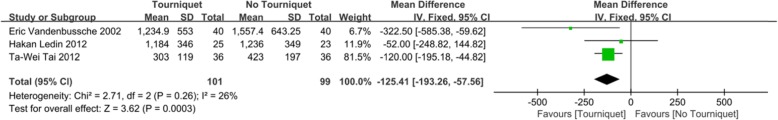
Forest plot for calculated blood loss

There were three studies [22, 24, 25] that met the inclusion criteria and provided data on calculated blood loss. Pooled data showed that calculated blood loss was significantly less in the tourniquet group (WMD = − 125.41 mL; 95% CI, − 193.26 to − 57.56; *n* = 200; *P* = 0.0003; *I*^2^ = 26%). The results are presented as a forest plot (Fig. 6).

**Fig. 7:**
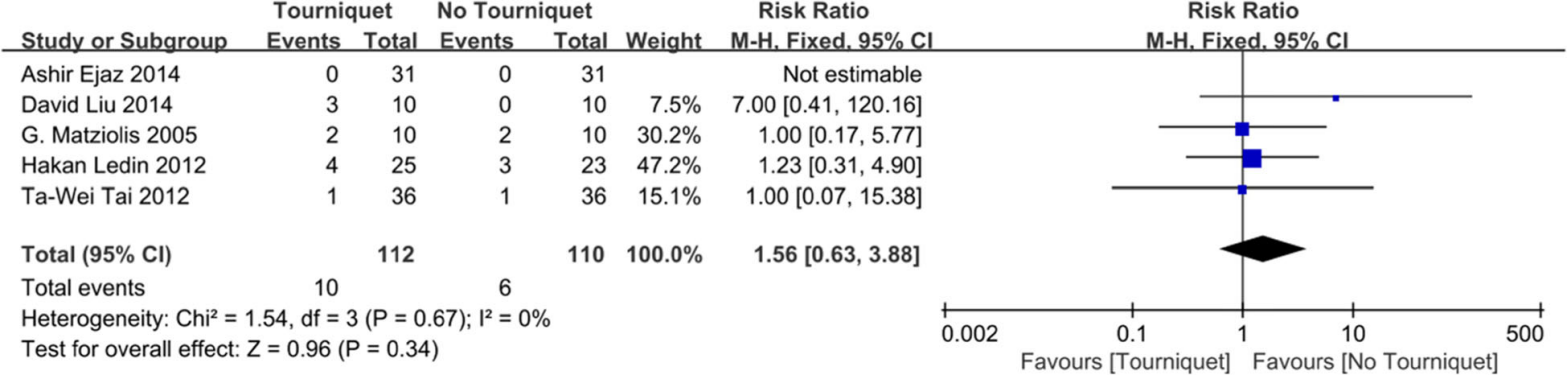
Forest plot for transfusion rate

There was no significant difference between the tourniquet and no tourniquet groups in terms of transfusion rate among five studies [18, 22, 25–27]. The pooled data (RR = 1.56; 95% CI, 0.63 to 3.88; *n* = 222; *P* = 0.34; *I*^2^ = 0%) are presented in a forest plot (Fig. 7).

**Fig. 8:**
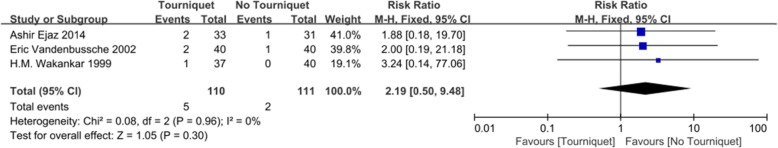
Forest plot for DVT rate

Three studies [23, 24, 27] provided data on the rate of DVT occurrence. However, any significant difference failed to be detected. The pooled data (RR = 2.19; 95% CI, 0.50 to 9.48; *n* = 221; *P* = 0.30; *I*^2^ = 0%) are presented in a forest plot (Fig. 8).

**Fig. 9:**
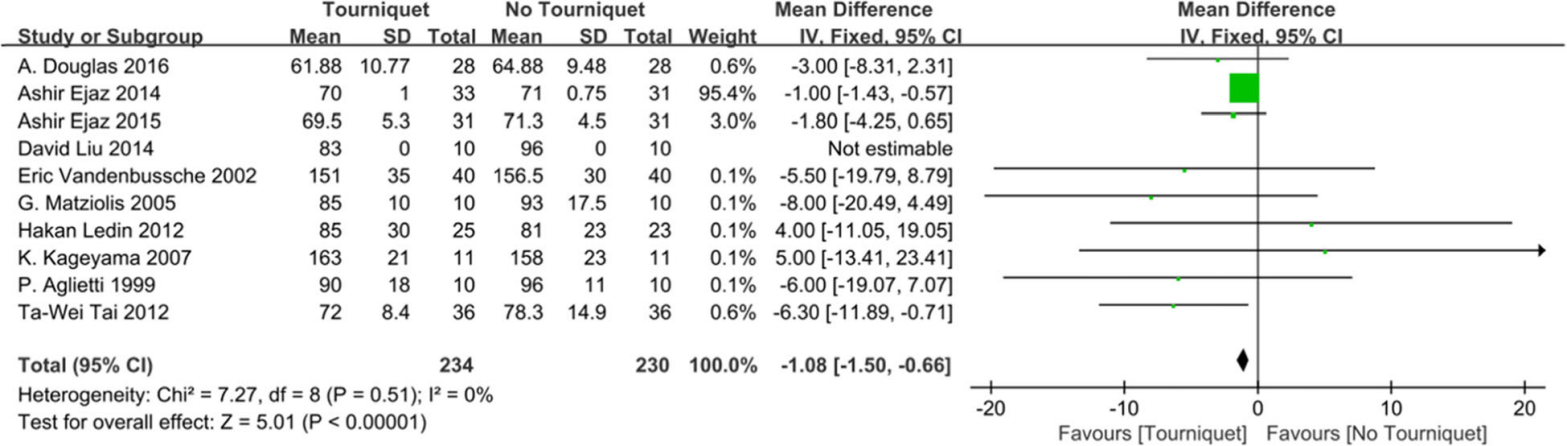
Forest plot for operation time

The operation time was able to be extracted from ten included studies [17–22, 24–27]. The forest plot of operation time showed that a significant difference existed between the two groups. Tourniquet use significantly decreased the operation time of TKA (WMD = − 1.08; 95% CI, − 1.50 to − 0.66; *n* = 464; *P* < 0.00001; *I*^2^ = 0%) (Fig. 9).

## Discussion

The main finding of the current meta-analysis was that using a tourniquet could significantly decrease the volume of intraoperative blood loss and the calculated blood loss. However, using a tourniquet did not significantly decrease postoperative blood loss, total blood loss, the rate of transfusion, or the rate of DVT in TKA surgery. The operation time with a tourniquet was significantly shorter than that without a tourniquet. However, the mean difference in operation time between using a tourniquet and not using a tourniquet was small.

The result showing that a tourniquet effectively reduced intraoperative blood loss was consistent with the result of previous meta-analyses [28, 29] (intraoperative blood loss: − 161.13 [− 203.52, − 118.74], 295 to 631 mL, 88.06–450.39, 279.82 to 116.60). Controlling intraoperative blood loss during TKA has at least two benefits: (1) it can provide a bloodless field of view and (2) it might be beneficial for bone cement to be able to penetrate bone trabeculae, which may contribute to increased stability of the prosthesis [30]. These benefits are the main reasons that surgeons choose to use tourniquets.

Furthermore, the necessity of a tourniquet is still debatable. Our meta-analysis confirmed that tourniquets can effectively decrease intraoperative blood loss but cannot decrease total blood loss. However, Alcelik et al. and Tai et al. [4, 31] found that the use of a tourniquet reduced the total blood loss by reducing intraoperative blood loss. However, some included studies in their analyses contained patients with rheumatoid arthritis, prosthesis revision, or osteonecrosis [32, 33]. Moreover, some included studies were nonrandomized control studies [34]. These confounders might result in bias and made the conclusions inconsistent with those of our study. In this meta-analysis, we only chose the studies that excluded these confounders, and we chose to include studies in which all patients underwent TKA for primary osteoarthritis. Therefore, the conclusions of our study should be more reliable.

### Postoperative drainage

Postoperative drainage volume might be influenced by many factors. The methods of drain use can significantly influence postoperative drainage volume. Stucinskas et al. and Shen et al. found the 4-h clamped drainage after TKA reduced the volume of postoperative drainage [35, 36].

Raleigh et al. concluded that clamping drains intermittently resulted in significantly less postoperative drainage than that observed with continuous suction drainage [37]. Park et al. found that intraarticular tranexamic acid administration plus 30-min drain clamping also significantly reduced postoperative drainage [38].

The use of a suction drain is another factor influencing postoperative drainage volume. According to Vandenbussche’s study, the blood drainage volume could reach 420–530 mL with the use of a suction drain [24]. Furthermore, according to the study by Aglietti et al., the blood drainage volume was only 290 mL without suction [19].

Our study found that the use of a tourniquet did not affect postoperative drainage volume. However, there were no similar patterns with the use of drainages in the included studies. Moreover, postoperative drainage might be affected by other factors, such as different hemostasis and anticoagulant methods. Therefore, the effect of a tourniquet on postoperative drainage volume needs to be further confirmed by more high-quality studies.

### Calculated blood loss

It was thought that calculated blood loss was more accurate than measured blood loss [39, 40]. Calculated blood loss contains recessive blood loss, which is believed to be caused by red blood cells entering the interstitial spaces [41]. The volume of calculated blood loss might be more than twice that of measured blood loss [13, 42]. Our study found that tourniquet use significantly reduced calculated blood loss. This outcome is different from previous findings. In contrast, Alcelik et al. thought that a tourniquet may slightly increase calculated blood loss, but there was no significant difference [4]. In addition, other studies had similar conclusions [28, 29, 43]. However, some included studies in the previous meta-analysis contained patients with rheumatoid arthritis, which might introduce major confounders to the results. According to Harvey’s study, the application of a tourniquet may increase fibrinolytic activity [32]. Rheumatoid arthritis patients might be more sensitive to tourniquet injuries, and their volume of postoperative blood loss might be more than that from ordinary patients. However, for patients with osteoarthritis, tourniquets can reduce the time of wound bleeding and reduce the extent of red blood cell infiltration into tissue space, reducing the amount of recessive blood loss, thereby reducing the calculated blood loss.

### Transfusion rate

Despite the fact that calculated blood loss and intraoperative blood loss were significantly different, our meta-analysis found no significant differences in the rate of transfusions between the tourniquet group and the no tourniquet group. This finding was consistent with the results of other meta-analyses. Tourniquets are not the only factor affecting blood transfusion rates. The blood transfusion standards in the included studies were not uniform and were influenced by the patient’s age, general physical conditions, and basic disease states. So, although the current findings are consistent, more high-quality studies are still needed to confirm whether tourniquets affect blood transfusion rates.

### Operation time

Our study confirmed that tourniquet use was effective in reducing operative time compared with no tourniquet use. A tourniquet can provide surgeons with a bloodless surgery field and facilitate the clear identification of anatomical structures during surgery, which might be conducive to shortening the operation time. Moreover, if the operation is performed without a tourniquet, more electrocoagulation and wound irrigation may be required during the operation. These additional procedures can increase the operation time. Zhang et al. determined that a tourniquet could reduce the operation time [28], which is consistent with our findings. However, it is worth noting that their study included patients with rheumatoid arthritis and patients undergoing revision surgery. These confounders might lead to biased results, because revision surgery requires additional surgical procedures, and rheumatoid patients might need more electrocoagulation. Our study excluded patients with rheumatoid arthritis and patients undergoing revisions, so the result of this meta-analysis is more reliable.

### Incidence of DVT

In this study, we found that the use of a tourniquet did not significantly increase the incidence of DVT. This finding was consistent with previous studies [29, 43, 44]. However, tourniquets are a potential risk factor for DVT. Parmet et al. found that the incidence of DVT with a tourniquet was 5.33 times higher than that with no tourniquet [45]. In addition, the incidence of DVT is affected by other factors, such as the administration of anticoagulant drugs, time until rehydration, and tourniquet pressure. In our meta-analysis, some included studies used anticoagulants [17, 19], and some did not use anticoagulants [18]. The same problem existed in other meta-analysis studies [44, 46]. These differences in inclusion criteria may lead to inconsistent results from different studies. Therefore, further high-quality studies are needed to confirm whether tourniquet use is an independent risk factor for DVT.

### Other complications

Because of insufficient data in the included literature, the current meta-analysis could not confirm whether there was a difference between using a tourniquet and not using a tourniquet in the incidence rates of some outcomes (i.e., wound hematoma, wound oozing, skin blistering, muscle injury, nerve palsy, and PE). The use of tourniquets might lead to more superficial infections of wounds [47]. However, Wakankar et al. determined that there was no difference between using tourniquets and not using tourniquets in terms of wound complications [23]. According to our study, 13 min was the longest time that was saved by the application of a tourniquet, and some RCTs found that a shorter operation time did not decrease the incidence of postoperative complications. Therefore, whether the shortening of operation time can decrease wound complications is still unclear at present [7, 29, 48]. Preoperative coexisting diseases, such as atherosclerosis, blood hypercoagulability, poor blood glucose, or uncontrolled blood pressure, were associated with the increasing incidence of postoperative complications. Tourniquets should be avoided in these patients, but we could not identify such patients from the current study. Patients with coexisting diseases in an RCT might affect the physician’s judgment on whether tourniquets cause certain complications [11]. Therefore, the current meta-analysis could not confirm whether the use of a tourniquet would increase the incidence of some surgical complications (i.e., wound hematoma, wound oozing, skin blistering, muscle injury, nerve palsy, and PE.)

### Limitations

The current meta-analysis has several limitations. Firstly, in some studies, the time for loosening the tourniquet was different. Some studies [19, 22] loosened the tourniquet before closing the incision, and some [24–26] loosened the tourniquet after applying the elastic bandage. Secondly, some studies differed in drainage patterns, hemostasis, and anticoagulation regimens, and this might result in a bias. Thirdly, in the included studies, none of the surgeons were blinded.

## Conclusions

According to the current meta-analysis, using a tourniquet may significantly decrease intraoperative blood loss, calculated blood loss, and the operation time, but tourniquet use did not significantly decrease the rate of blood transfusion or the rate of DVT in TKA. More research is needed to determine if there are fewer complications without the use of tourniquets in TKA surgery.

## Data Availability

Data sharing is not applicable to this article as no datasets were generated or analyzed during the current study.

## Abbreviations

CI: Confidence interval
SD: Standard deviation
DVT: Deep vein thrombosis
PE: Pulmonary embolism
RCTs: Randomized controlled trials
TKA: Total knee arthroplasty
WMD: Weighted mean difference
